# Supplementary figures and images for: Hemodynamic outcomes in patients undergoing bidirectional cavopulmonary connection with additional or antegrade pulmonary blood flow: a single-centre retrospective study
Source: PeerJ. 2025 Oct 6;13:e20021. doi: 10.7717/peerj.20021 (PMC12510245; doi:10.7717/peerj.20021)

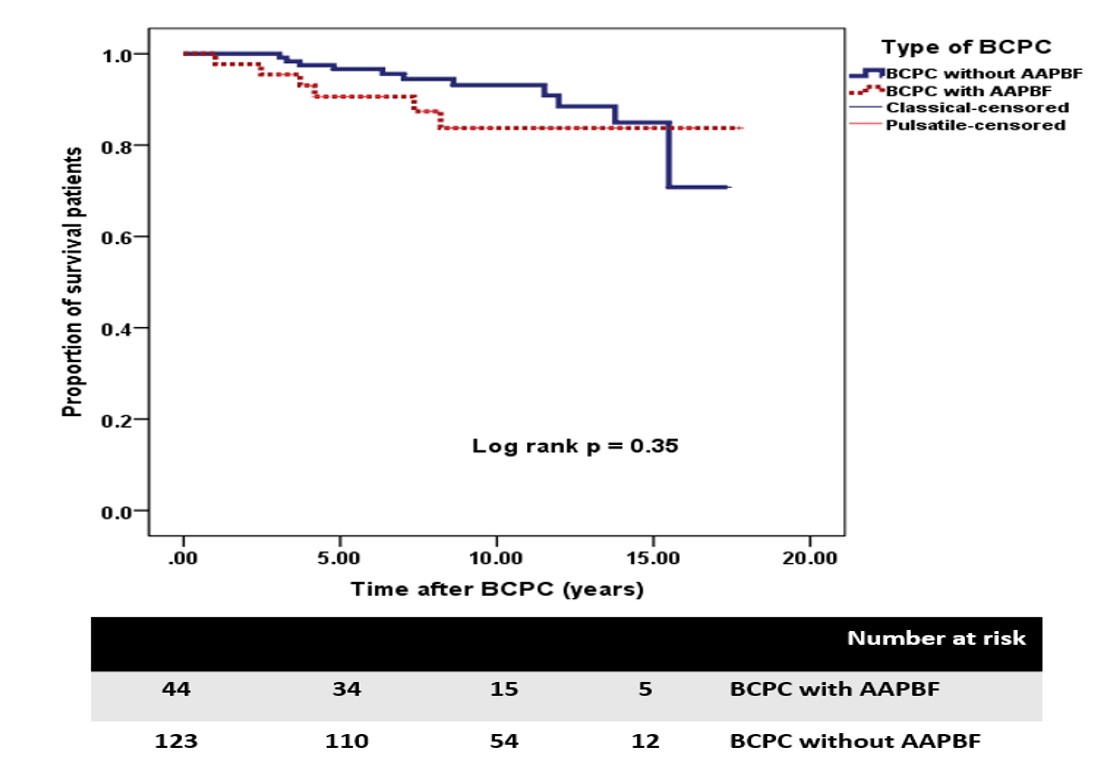

Supplement: Supplemental Information 2 — BCPC, bidirectional cavopulmonary connection; AAPBF, additional or antegrade pulmonary blood flow [file peerj-13-20021-s002.jpg]
