# Supplementary material for: Hemodynamic outcomes in patients undergoing bidirectional cavopulmonary connection with additional or antegrade pulmonary blood flow: a single-centre retrospective study
Source: PeerJ. 2025 Oct 6;13:e20021. doi: 10.7717/peerj.20021 (PMC12510245; doi:10.7717/peerj.20021)
Supplement: Supplemental Information 3 [file peerj-13-20021-s003.docx]

**Supplementary table files**

**Table S1** Comparison of changes in Z-scores of RPA, LPA at pre-BCPC and pre-TCPC cardiac catheterization (n=167)

|  | **AAPBF**  **(n = 44)** | | | **Without AAPBF**  **(n = 123)** | | |
| --- | --- | --- | --- | --- | --- | --- |
|  | Pre-BCPC | Pre-TCPC | *p*-value | Pre-BCPC | Pre-TCPC | *p*-value |
| Z score RPA | 1.91  (0.37-3.74) | 2.45  (1.11-4.05) | 0.616 | 1.69  (0.54-3.26) | 1.06  (-0.05-1.78) | **<0.001*** |
| ∆RPA Z score | 0.44 (-0.96-1.8) | | | -0.94 (-2.45-0.32) | | **0.001*** |
| Rate RPA Z score change/month | 0.0064 (-0.271-0.325) | | | -0.0178 (-0.0414-0.0043) | | **0.005*** |
| Z score LPA | 1.15  (-0.24-2.93) | 1.17  (-0.56-2.39) | 0.757 | 1.29  (0.12-2.99) | -0.06  (-0.95-0.93) | **<0.001*** |
| ∆LPA Z score | -0.010 (-1.87-1.22) | | | -1.435 (-2.735- -0.170) | | **0.001*** |
| Rate LPA Z score change/month | -0.0001 (-0.0343-0.0261) | | | -0.0190 (-0.0393- -0.0032) | | **0.003*** |

Data represented as mean ± SD, median (range), and n (% within column).

* Statistical significance at p-value <0.05

*AAPBF*, additional or antegrade pulmonary blood flow; *BCPC*, bidirectional cavopulmonary connection; *TCPC*, total cavopulmonary connection; *RPA*, right pulmonary artery; *LPA*, left pulmonary artery

**Table S2** Comparison of mean differences of RPA, LPA size at pre-BCPC and pre-TCPC cardiac catheterization with baseline adjustment using ANCOVA statistics

|  | **Group 1 with AAPBF**  **(95%CI)** | **Group 2 without AAPBF (95%CI)** | **Mean difference**  **(95%CI)** | **Cohen’s d** | ***p*-value** |
| --- | --- | --- | --- | --- | --- |
| RPA (mm) | 13.19  (12.41-13.97) | 11.26  (10.80-11.72) | 1.93  (1.02-2.84) | 0.098 | <0.001** |
| LPA (mm) | 11.44  (10.61-12.26) | 10.00  (9.53-10.51) | 1.42  (0.45-2.38) | 0.049 | 0.004** |
| McGoon ratio | 2.27  (2.13-2.41) | 2.01  (1.93-2.01) | 0.26  (0.09-0.42) | 0.058 | 0.002*** |
| Nakata index (mm^2^/m^2^) | 318.01  (285.20-350.97) | 235.18  (215.72-254.65) | 82.90  (44.37-121.44) | 0.101 | <0.001** |

* Statistical significance at *p*-value <0.05 ** Statistical significance at *p*-value <0.017 (Bonferroni correction) ***Statistical significance at *p*-value <0.025 (Bonferroni correction)

*AAPBF*, additional or antegrade pulmonary blood flow; *CI,* confidence interval; *RPA*, right pulmonary artery; *LPA*, left pulmonary artery

**Table S3** Details of atrioventricular valve progression

|  | **Group 1 with AAPBF (n = 44)** | **Group 2 without AAPBF (n=123)** | ***p*-value** |
| --- | --- | --- | --- |
| **Initial moderate to severe AVVR**  - Remain moderate to severe  - Improved to mild | 4 (9.1%)  2 (50%)  2 (50%) | 17 (13.8%)  7 (5.7%)  10 (8.1%) | 0.417  1.000  0.734 |
| **Final moderate to severe AVVR**  - Progress from initially mild | 6 (13.6%)  4 (9.1%) | 20 (16.3%)  8 (6.5%) | 0.638  0.518 |
| **Type of AVV:**  Common  TV or MV | 5 (83.3%)  1 (16.7%) | 12 (60%)  8 (40%) | 0.380 |
| Heterotaxy | 4 (66.7%) | 12 (60%) | 1.000 |
| **Dominant ventricle:**  Right  Left  Unidentified | 1 (16.7%)  -  5 (83.3%) | 9 (45%)  3 (15%)  8 (40%) | 0.170 |
| **Previous operation:**  Shunt  PA banding  Norwood  No previous operation | -  1 (16.7%)  -  5 (83.3%) | 6 (30%)  7 (35%)  1 (5%)  6 (30%) | 0.134 |
| **Ventricular function:**  Good  Reduced | 5  1 | 17  3 | 1.000 |
| Arrhythmia | 2 | 8 | 1.000 |

* Statistical significance at *p*-value <0.05

*AAPBF*, additional or antegrade pulmonary blood flow; *AVV*, atrioventricular valve; *AVVR,* atrioventricular valve regurgitation; *TA*, tricuspid; *MV,* mitral valve; *PA,* pulmonary artery
