# Supplementary material for: Hemodynamic outcomes in patients undergoing bidirectional cavopulmonary connection with additional or antegrade pulmonary blood flow: a single-centre retrospective study
Source: PeerJ. 2025 Oct 6;13:e20021. doi: 10.7717/peerj.20021 (PMC12510245; doi:10.7717/peerj.20021)
Supplement: Supplemental Information 5 — Graphical abstract showed the method of the study and outcomes. The graphs shown significant growth of RPA size, LPA size and Nakata index of patients who underwent pulsatile bidirectional Glenn operation with additional antegrade pulmonary blood flow. [file peerj-13-20021-s005.pdf]

# Hemodynamic outcomes of patient undergoing bidirectional Glenn operation with additional antegrade pulmonary blood flow

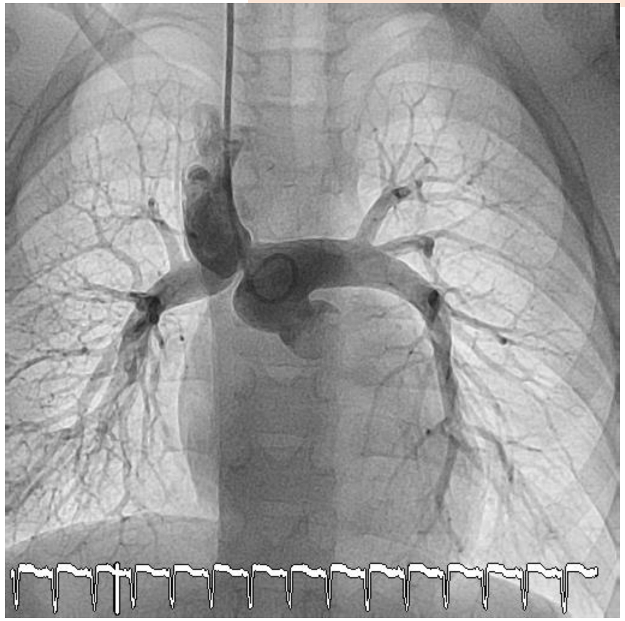

BCPC with APBF

**Methods**: Single center, retrospective review 167 patients who underwent BCPC between 2006 to 2022.

- BCPC (n=123)
- BCPC with AAPBF (n=44)

- AAPBF provided **significant** growth of both pulmonary artery branches and prevent negative remodeling of the McGoon ratio and Nakata index.
- AAPBF **did not impact** on mean pulmonary artery pressure, ventricular end diastolic pressure, pulmonary vascular resistance index and survival.

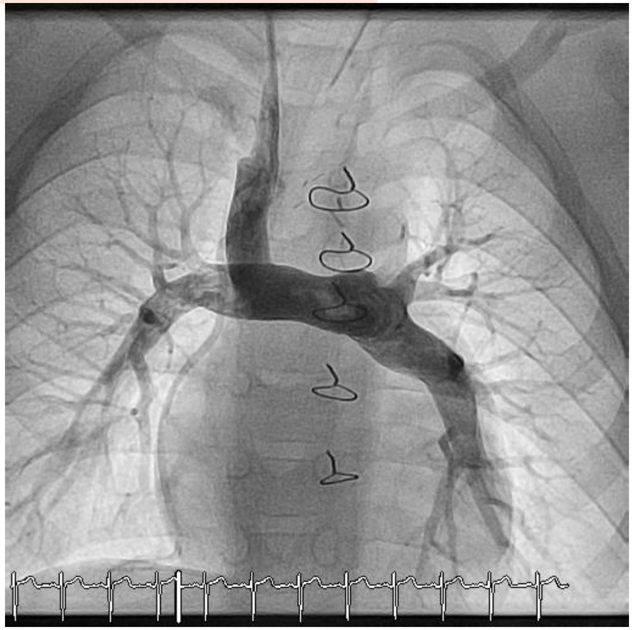

Conventional BCPC

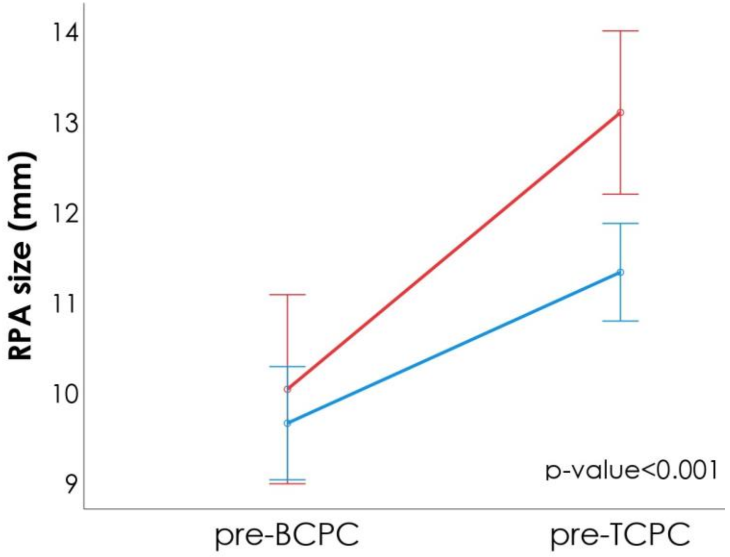


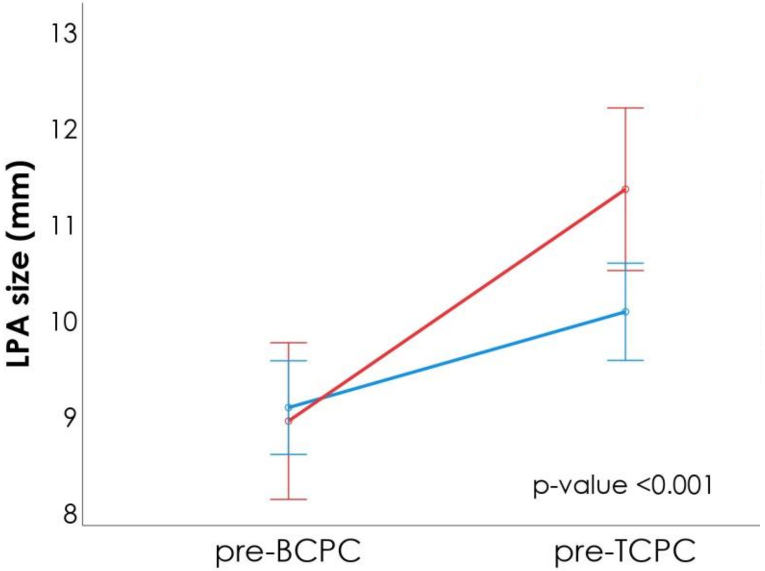


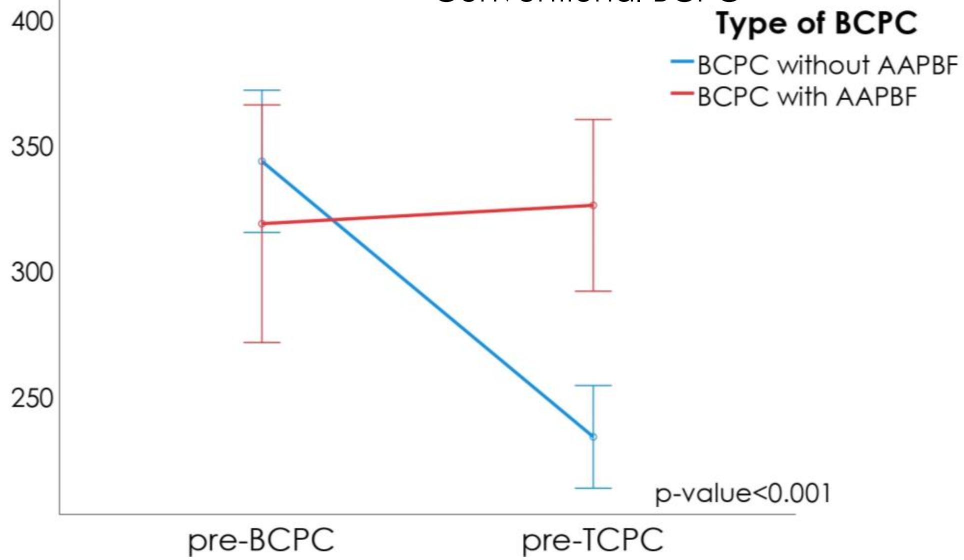


*BCPC: bidirectional cavopulmonary connection; TCPC: total cavopulmonary connection; AAPBF: additional or antegrade pulmonary blood flow; RPA: right pulmonary artery; LPA: left pulmonary artery
